# Supplementary material for: A selective inhibitor of ceramide synthase 1 reveals a novel role in fat metabolism
Source: Nat Commun. 2018 Aug 21;9:3165. doi: 10.1038/s41467-018-05613-7 (PMC6104039; doi:10.1038/s41467-018-05613-7)
Supplement: Supplementary file 1 — Supplementary Information [file 41467_2018_5613_MOESM1_ESM.pdf]

Supplementary Information for

A selective inhibitor of ceramide synthase 1 reveals a novel role in fat metabolism

Turner *et al.*

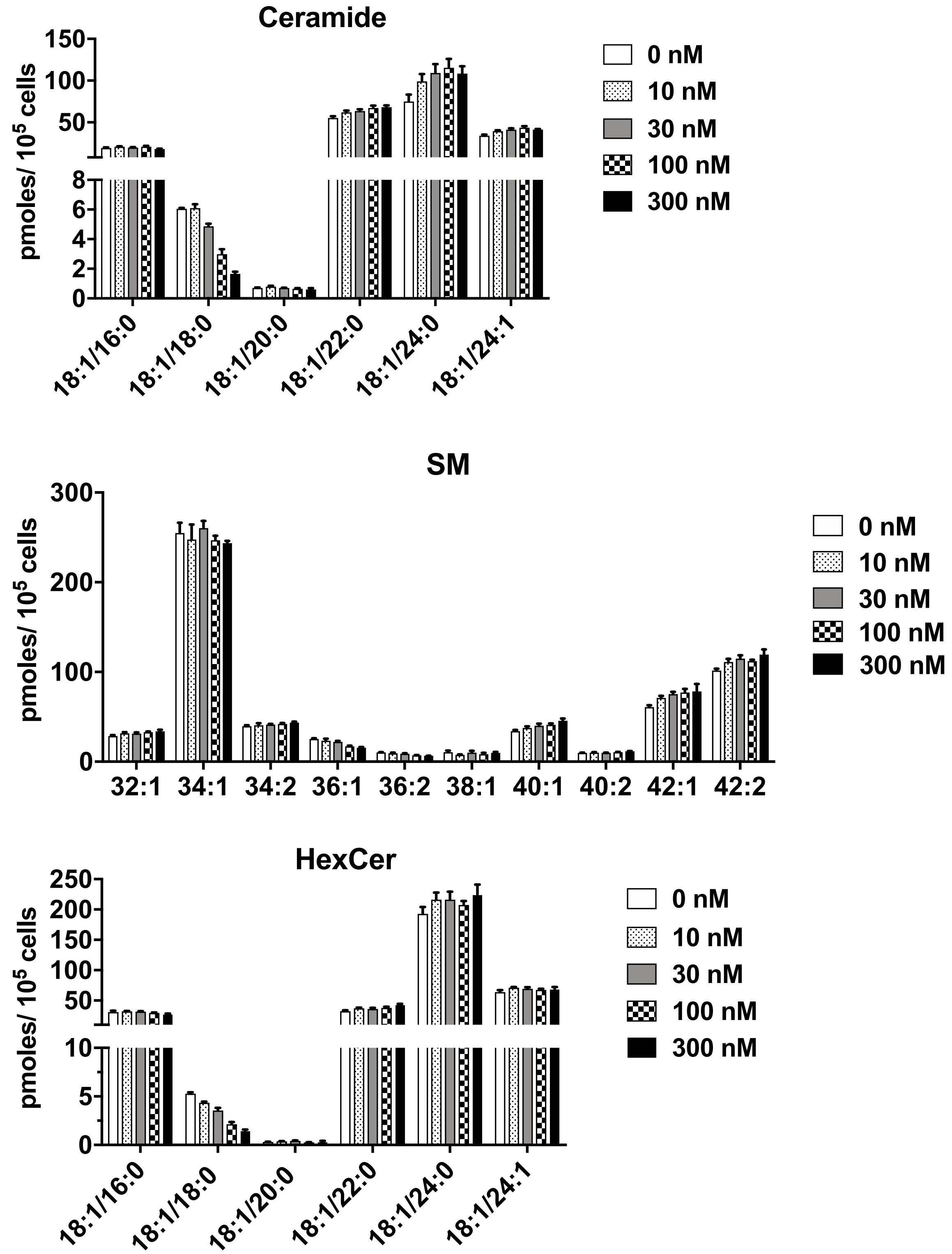

Supplementary Figure 1. Ceramide, SM, and HexCer levels in HEK293 cells treated with P053. Absolute lipid levels were determined by targeted LC-MS/MS, relative to external standard curves. Results are mean ± SEM (n = 4) from one of two identical experiments. Statistically significant results, based on two independent experiments, are as indicated in Figure 2b-d. Raw data for these figures is provided in Supplementary Data File 1.

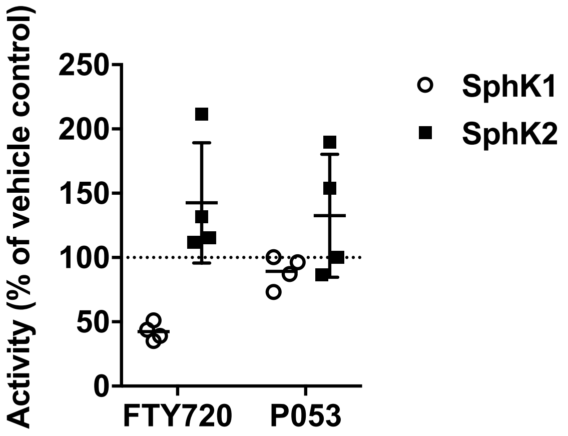

Supplementary Figure 2. P053 does not inhibit sphingosine kinases. Activity of recombinant human sphingosine kinases 1 and 2 (SphK1 and 2) was assessed in the presence of 10 μM FTY720 or P053 (four assays). Activity is expressed as a percentage of the vehicle control.

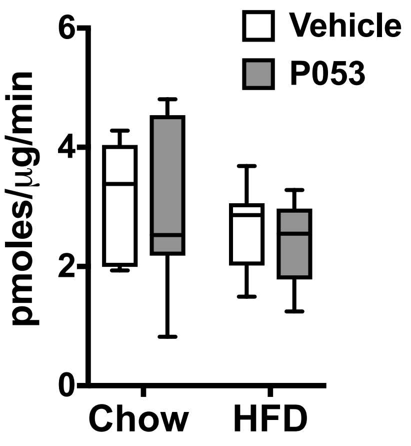

Supplementary Figure 3. C24 ceramide synthase activity in SkM of P053-treated mice. C24 ceramide synthase activity of SkM from mice treated for 4 weeks with P053 or vehicle control. N = 10 for all groups except chow + P053 group, where n = 8. Two-way ANOVA revealed no significant effect of diet (P = 0.1132) or P053 treatment (P = 0.4931) on C24 ceramide synthase activity.

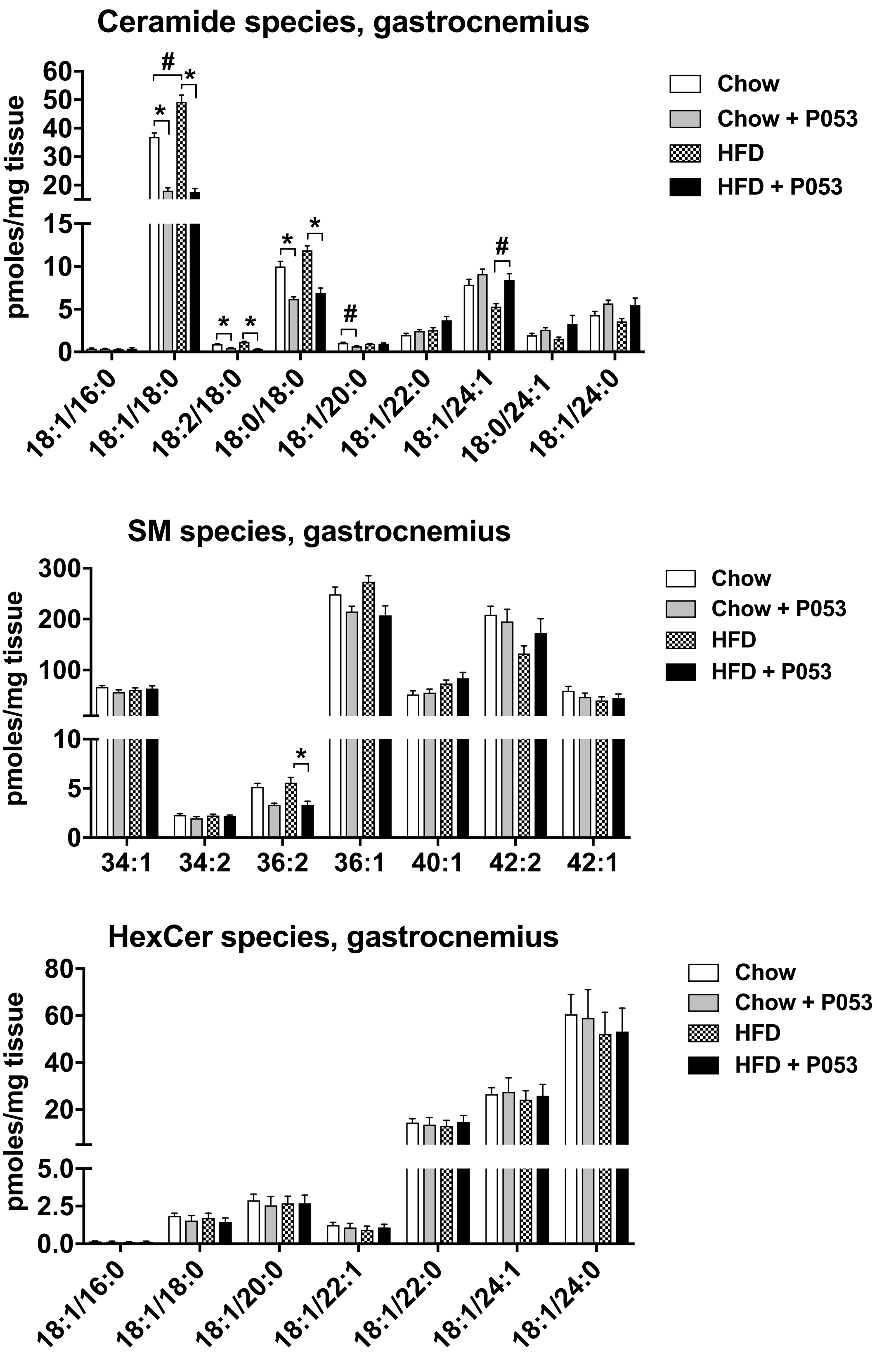

Supplementary Figure 4. Ceramide, SM, and HexCer content of gastrocnemius muscle. Lipids levels were quantified by targeted LC-MS/MS. Results are mean ± SEM, n = 10 for all groups; #, P < 0.01; *, P < 0.001, as determined by two-tailed t-tests adjusted for multiple comparisons. Raw data for these figures is provided in Supplementary Data File 1.

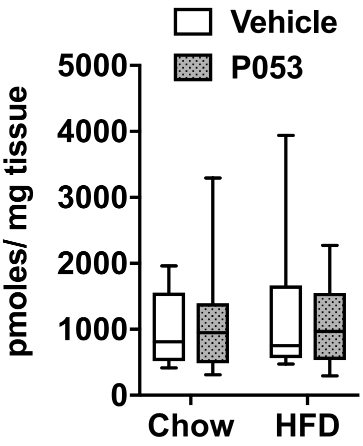

Supplementary Figure 5: P053 does not affect diacylglycerol levels. Total diacylglycerol in SkM, n = 20 for all groups except Chow + P053, where n = 18 and HFD + P053, where n = 19. DAG levels were determined by lipidomic profiling on a QExactive Plus. Two-way ANOVA revealed no significant effect of either diet (P = 0.55) or P053 (P = 0.96).

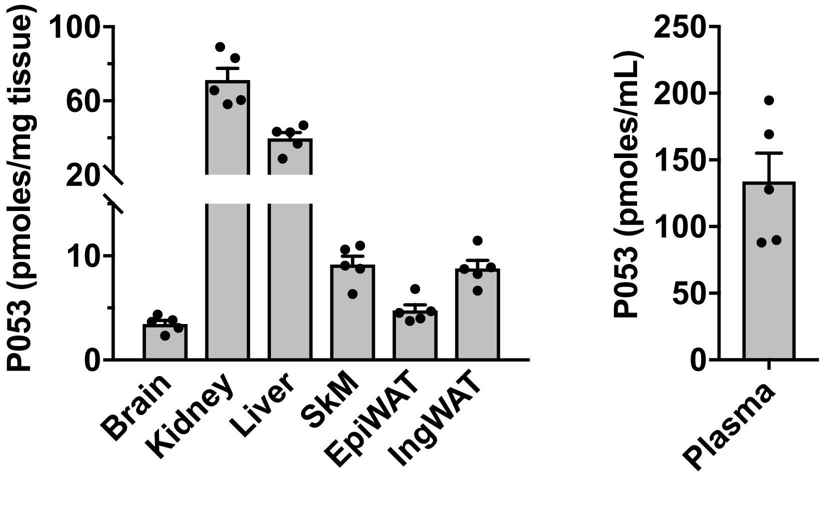

Supplementary Figure 6. P053 accumulation in various tissues. P053 was quantified, using LC-MS/MS, in multiple tissues of mice administered 5 mg/kg P053 daily for 2 weeks (n = 5 mice). No P053 was detected in mice administered vehicle only.

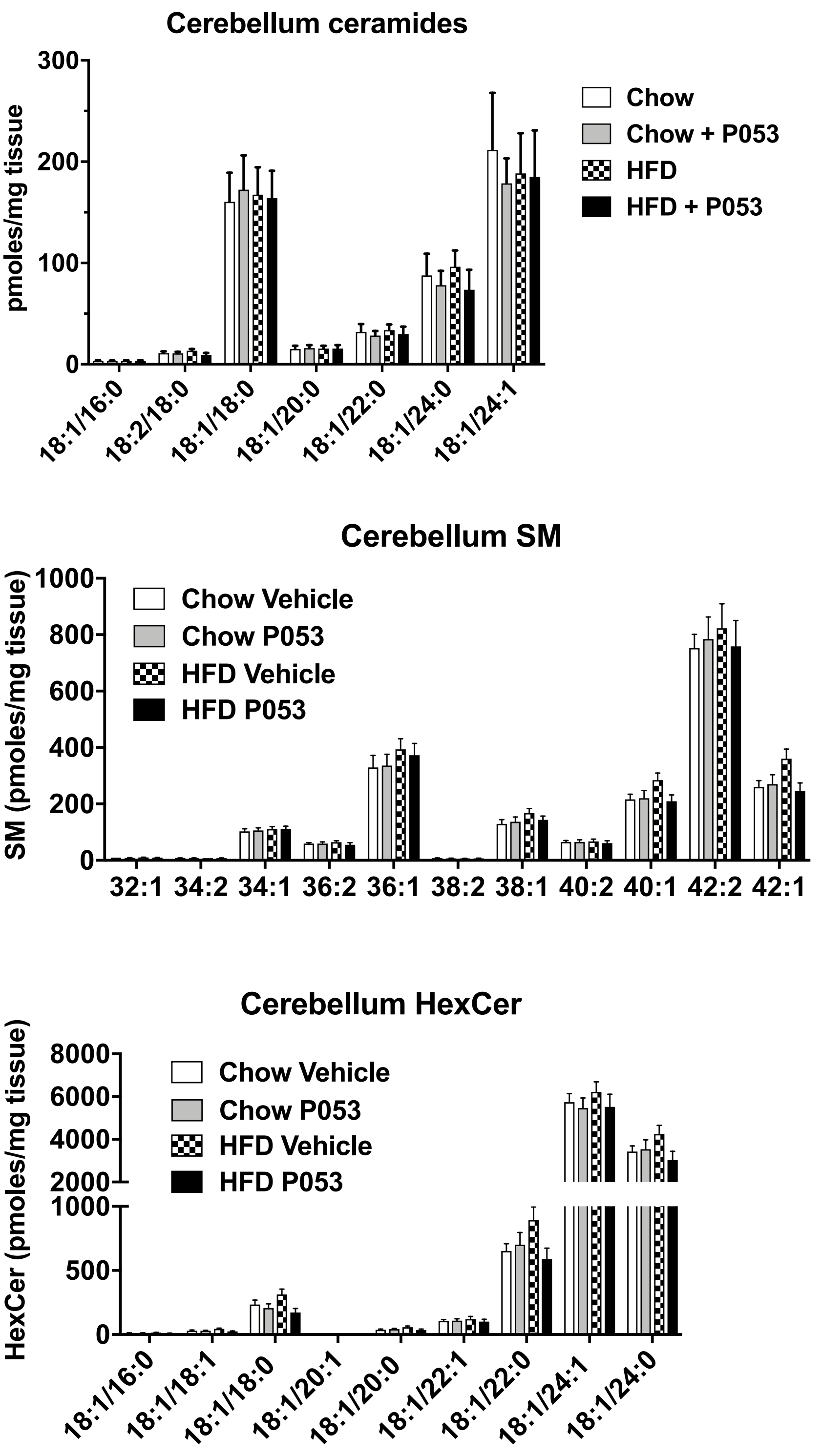

Supplementary Figure 7. P053 does not affect brain ceramides. Ceramide, SM, and HexCer levels in the cerebellum of vehicle- or P053-treated mice on a chow or HFD. N = 10 mice per group, except for chow vehicle group (n = 9). Mean ± SEM shown. No lipids were significantly affected by diet or by P053, as determined by t-tests adjusted for multiple comparisons. C18:0 HexCer was 45% lower in the P053-treated compared to the vehicle-treated HFD group (unadjusted P value = 0.02; adjusted value P = 0.16). Raw data for these figures is provided in Supplementary Data File 1.

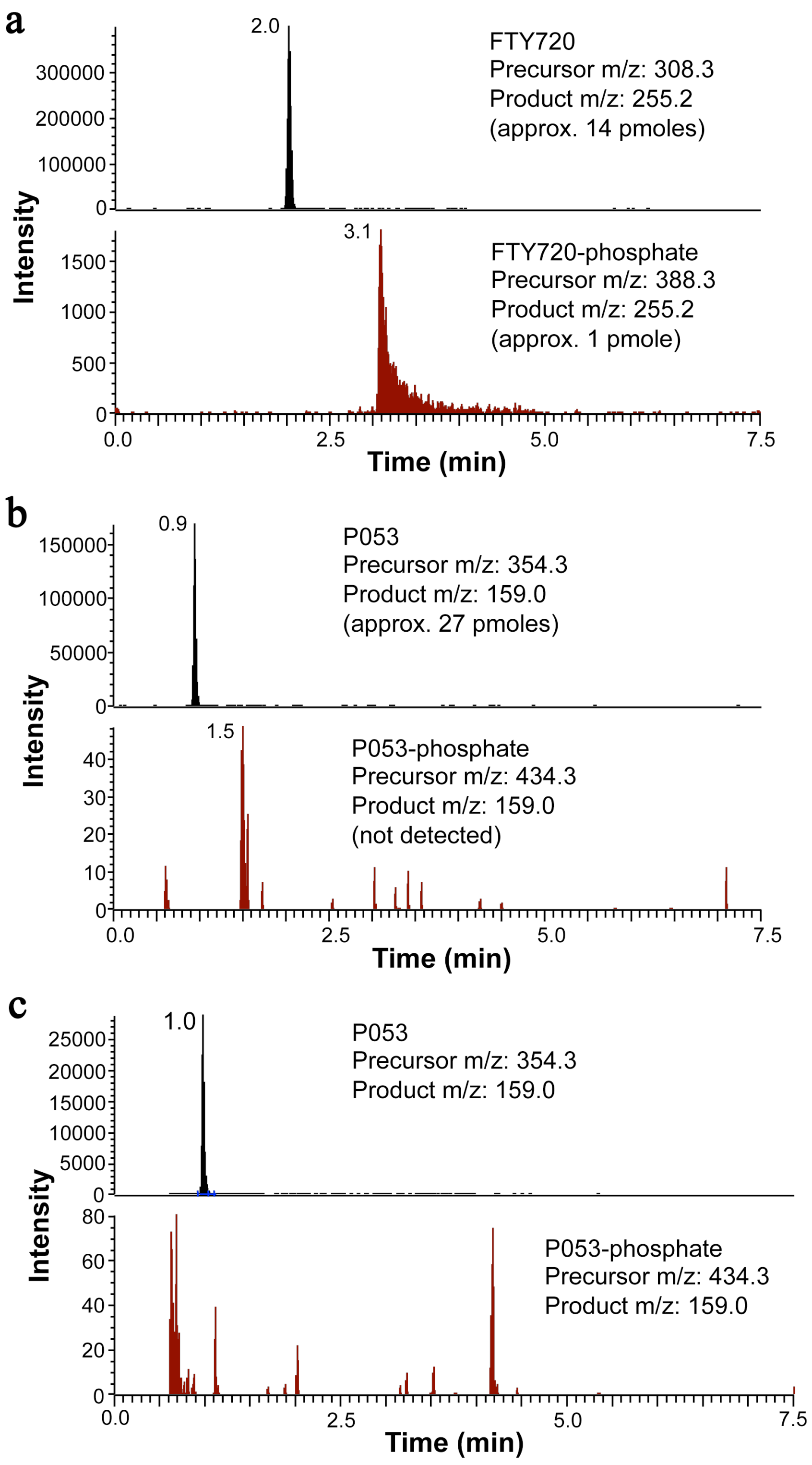

Supplementary Figure 8. P053 is not phosphorylated *in situ* or *in vivo*. (a-b) HEK293 cells were incubated for 2 h with 1 μM FTY720 or P053, then subjected to lipid extraction and LC-MS/MS analysis. Chromatograms for (a) FTY720 and its phosphate, and (b) P053 and its putative phosphate are shown. Note the MS intensity counts (y-axis) show a definitive peak for FTY720-phosphate, whereas there is only background signal in the case of P053-phosphate. (c) A clear peak for P053 is detected in plasma of mice administered the drug at 5 mg/kg/day for 6 weeks, corresponding to a plasma concentration of ~300 nM. No peak is detected for P053-phosphate (again, only background signal is detected).

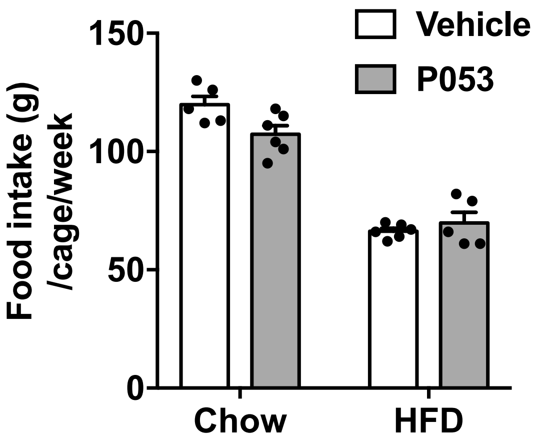

Supplementary Figure 9: Food intake is not affected by P053 treatment. Weekly food intake per cage of 4 mice, monitored on 5-6 independent occasions over the course of the P053 treatment period. Two-way ANOVA revealed a significant effect of diet ($P < 0.0001$) but not P053 treatment ($P = 0.1483$) on food intake.

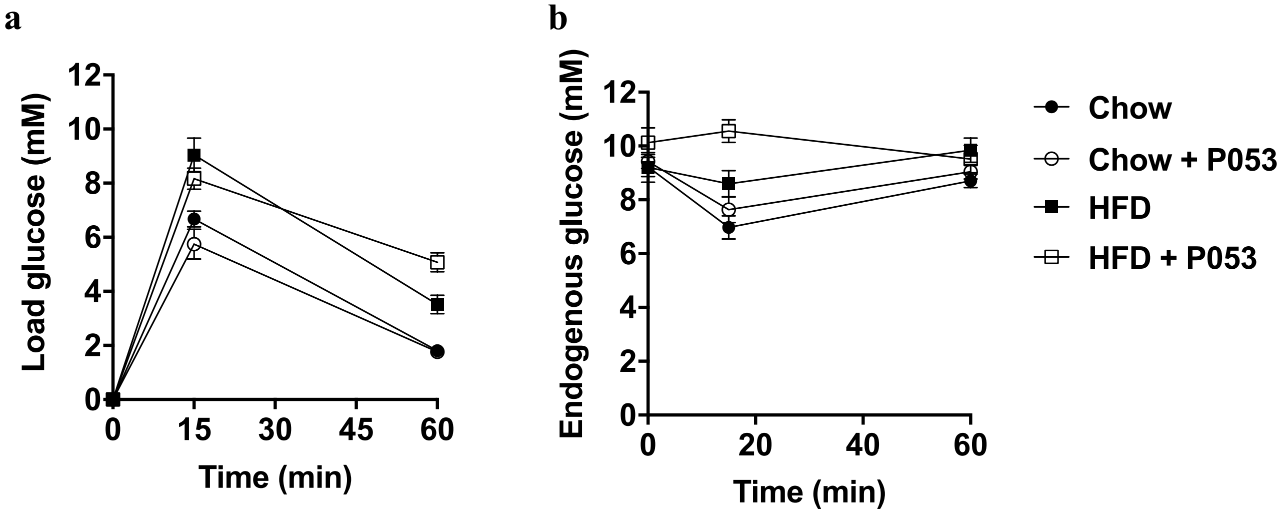

Supplementary Figure 10. P053 does not affect diet-induced changes in glucose disposal or endogenous glucose synthesis. Plasma concentration of (a) labelled glucose resulting from an oral glucose load, and (b) endogenous glucose, during the oral glucose tolerance test. Mice were fed chow or HFD with vehicle or 5 mg/kg P053 ($n = 10$ per group). Two-way ANOVA analysis of the iAUC for the labelled glucose load (panel a) showed a highly significant effect of diet ($P < 0.0001$) with no effect of P053 treatment ($P = 0.71$).

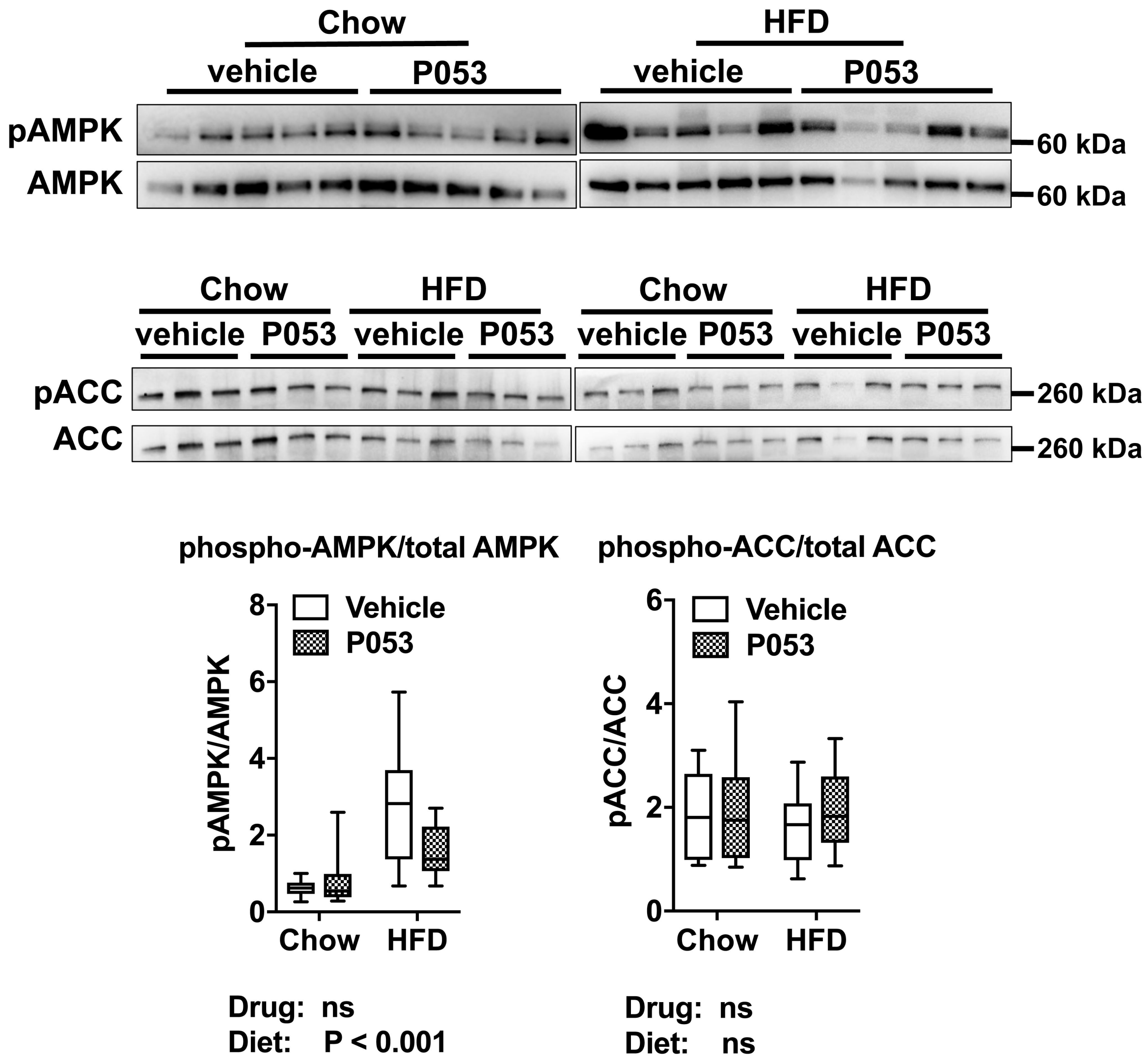

Supplementary Figure 11: P053 treatment does not promote AMPK phosphorylation. Western blots for phospho-AMPK (Thr172), total AMPK, phospho-ACC (Ser79), and total ACC in vehicle- or P053-treated mice on a chow or HFD. Denistometry for phospho-AMPK/total AMPK and phospho-ACC/total ACC is shown (n = 10 mice per group).

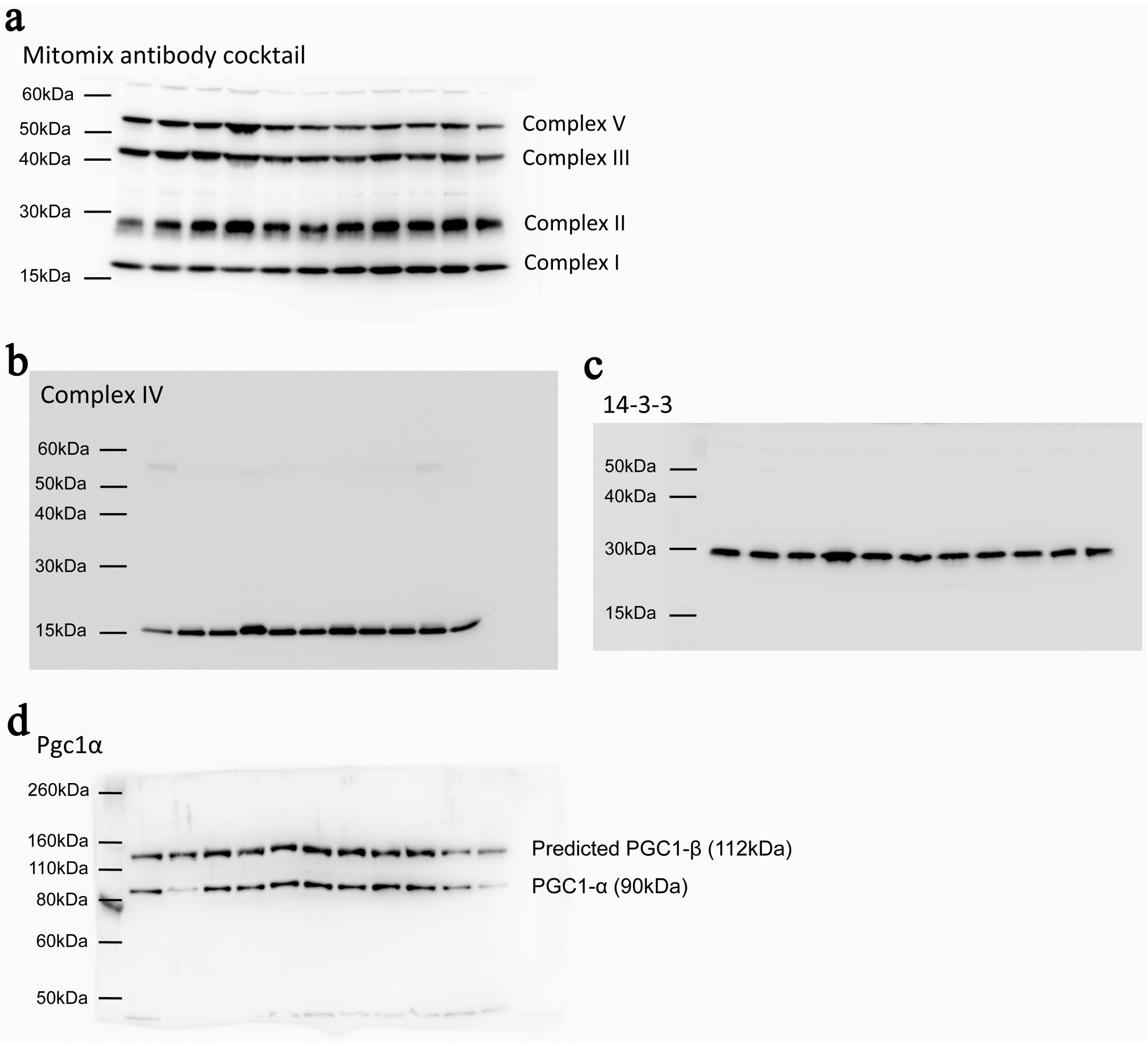

Supplementary Figure 12: Full membrane images for blots in Figure 6. (A) mitomix antibody cocktail, (B) mitochondrial complex IV, (C) 14-3-3 protein, and (D) Pgc-1α. Position of molecular weight markers is shown on the right. Membranes in a, b, and c were cut to facilitate simultaneous blotting of high molecular weight proteins.
