# Supplementary material for: A selective inhibitor of ceramide synthase 1 reveals a novel role in fat metabolism
Source: Nat Commun. 2018 Aug 21;9:3165. doi: 10.1038/s41467-018-05613-7 (PMC6104039; doi:10.1038/s41467-018-05613-7)
Supplement: Supplementary file 3 — Description of Additional Supplementary Information [file 41467_2018_5613_MOESM3_ESM.docx]

**Description of Additional Supplementary Files**

File Name: Supplementary Data 1

Description:

Raw data from all bar graphs in the main figures and supplementary figures.

File Name: Supplementary Data 2

Description:

Lipidomic data from muscle and liver
